# Implementing a text message-based intervention to support type 2 diabetes medication adherence in primary care: a qualitative study with general practice staff

**DOI:** 10.1186/s12913-023-09571-9

**Published:** 2023-06-10

**Authors:** Karen Butler, Yvonne Kiera Bartlett, Nikki Newhouse, Andrew Farmer, David P. French, Cassandra Kenning, Louise Locock, Rustam Rea, Veronika Williams, Jenny Mc Sharry

**Affiliations:** 1 Health Behaviour Change Research Group, School of Psychology, University of Galway, University Road, Galway, Ireland; 2 Manchester Centre for Health Psychology, School of Health Sciences, University of Manchester, Manchester, UK; 3 Nuffield Department of Primary Care Health Sciences, University of Oxford, Oxford, UK; 4 Division of Population Health, Health Services Research & Primary Care, School of Health Sciences, University of Manchester, Manchester, UK; 5 Health Services Research Unit, University of Aberdeen, Aberdeen, UK; 6 Oxford Centre for Diabetes, Endocrinology and Metabolism, NIHR Oxford Biomedical Research Centre, Oxford University Hospitals NHS Foundation Trust, Oxford, UK; 7 Faculty of Education and Professional Studies, School of Nursing, Nipissing University, North Bay, Canada

**Keywords:** Implementation science, Text message-based intervention, Primary care, General practice, Medication adherence, Diabetes

## Abstract

**Background:**

The Support through Mobile Messaging and digital health Technology for Diabetes (SuMMiT-D) project has developed, and is evaluating, a mobile phone-based intervention delivering brief messages targeting identified behaviour change techniques promoting medication use to people with type 2 diabetes in general practice. The present study aimed to inform refinement and future implementation of the SuMMiT-D intervention by investigating general practice staff perceptions of how a text message-based intervention to support medication adherence should be implemented within current and future diabetes care.

**Methods:**

Seven focus groups and five interviews were conducted with 46 general practice staff (including GPs, nurses, healthcare assistants, receptionists and linked pharmacists) with a potential role in the implementation of a text message-based intervention for people with type 2 diabetes. Interviews and focus groups were audio-recorded, transcribed and analysed using an inductive thematic analysis approach.

**Results:**

Five themes were developed. One theme ‘The potential of technology as a patient ally’ described a need for diabetes support and the potential of technology to support medication use. Two themes outlined challenges to implementation, ‘Limited resources and assigning responsibility’ and ‘Treating the patient; more than diabetes medication adherence’. The final two themes described recommendations to support implementation, ‘Selling the intervention: what do general practice staff need to see?’ and ‘Fitting the mould; complementing current service delivery’.

**Conclusions:**

Staff see the potential for a text message-based support intervention to address unmet needs and to enhance care for people with diabetes. Digital interventions, such as SuMMiT-D, need to be compatible with existing systems, demonstrate measurable benefits, be incentivised and be quick and easy for staff to engage with. Interventions also need to be perceived to address general practice priorities, such as taking a holistic approach to care and having multi-cultural reach and relevance. Findings from this study are being combined with parallel work with people with type 2 diabetes to ensure stakeholder views inform further refinement and implementation of the SuMMiT-D intervention.

**Supplementary Information:**

The online version contains supplementary material available at 10.1186/s12913-023-09571-9.

## Background

Type 2 diabetes is a lifelong condition characterised by the ineffective use of insulin, and accounts for the majority of people with diabetes around the world [1]. Approximately 4.7 million people in the UK are currently living with a type 2 diabetes diagnosis [2]. The global economic cost of diabetes has been projected to increase from U.S. $1.3 trillion in 2015 to $2.2 trillion in 2030 [3] and reflects the health risk posed by type 2 diabetes complications such as cardiovascular disease, stroke, renal failure and retinopathy [1, 4, 5].

Although type 2 diabetes poses significant health risks, the risks of complications from the disease can be reduced through self-management behaviours such as healthy diet and exercise, and medication adherence [6]. Despite this, many people with type 2 diabetes report concerns about medications or difficulty taking them as prescribed [7]. Non-adherence to diabetes medications can lead to poor diabetes control and poor outcomes [8], and in the UK is estimated to cost £100 million annually in avoidable treatment costs [9]. Although many interventions to improve adherence to medications by people with type 2 diabetes exist, approaches have yet to be standardised or delivered at scale, resulting in inconsistent outcomes [10].

Digital interventions have demonstrated promising results in altering a wide range of health-related behaviours. Text messaging is a low cost digital intervention with a wide reach given the near universal adoption of mobile phones. Text messaging does not require recipients to pay for internet use and is the primary means currently used in UK general practice to communicate appointment reminders and to support prescription management. Systematic review evidence has suggested that text message-based interventions can increase medication adherence across chronic conditions, although the identification of the features of text message interventions that improve success has been highlighted as requiring further investigation [11]. Specific to type 2 diabetes, evidence has suggested that interventions based on messaging and monitoring have the potential to improve medication adherence [12]. Digital interventions are promising candidates for widespread implementation as they are low cost, can be delivered at scale within existing infrastructure, and can be easily and cheaply modified. The incorporation of digital interventions within standard care may be particularly timely given the shift towards remote monitoring and reduced in-person contact during the COVID-19 pandemic. However, a review of studies of brief messages to support medication adherence in type 2 diabetes found limitations with existing brief messaging interventions, including inadequate detail on the content of messages and how the messages were developed and limited use of theoretical frameworks in the development of brief messaging interventions [12].

Based on the potential for digital innovation within diabetes healthcare, a programme of research has explored the systematic development of the SUpport through Mobile Messaging and digital health Technology for Diabetes (SuMMiT-D) intervention, a system for delivering automated, brief messages to support medication adherence for type 2 diabetes [13]. The SuMMiT-D intervention was designed to target a broad range of individuals with type 2 diabetes and to support people in both the initiation and implementation phases of medication adherence. The SuMMiT-D intervention aims to address limitations with existing text message-based interventions by taking a theory and evidence-based approach to intervention development, drawing on the perspectives and preferences of people with type 2 diabetes in refining message content, and clearly specifying the content of messages included in the intervention [14]. The proposed intervention contains over 300 unique messages based on established behaviour change techniques [15] and has demonstrated good acceptability among people with type 2 diabetes [16].

Alongside efficacy and acceptability to people with type 2 diabetes, the impact of digital interventions such as SuMMiT-D are dependent on integration and uptake in primary care. Difficulties translating evidence-based interventions from a trial context to routine clinical care are well-documented, a phenomenon known as the “evidence to practice gap” [17]. Primary care settings are particularly susceptible to this phenomenon, largely due to complexity of the setting [17]. Primary care is typically characterised by multiple interactions involving patients, GPs, pharmacists, and administrative staff, each of whom have their own concerns and priorities. This makes primary care a difficult context in which to implement interventions, since successful knowledge translation relies on change at multiple levels.

The SuMMiT-D intervention is being delivered in the UK where primary diabetes care is largely carried out through general practice. This study aims to explore general practice staff perceptions of the implementation of text message-based support for medication adherence within current and future diabetes care. Exploring anticipated implementation during the initial development of the SuMMiT-D intervention will facilitate both identification of the actions required for future implementation and inform further development of the intervention to make it appealing to key general practice stakeholders.

## Methods

### Design

A semi-structured qualitative study, with data collection through focus groups and supplementary interviews, was conducted. Focus groups were chosen to facilitate multi-disciplinary discussion and were conducted following the initial development of the SuMMiT-D intervention. Supplementary interviews were conducted to further explore issues identified during focus groups and to explore if additional insights would be identified from continued from data collection. Ethical approval for this study was provided by the NHS National Research Ethics Committee in April 2017 and all participants provided informed consent in writing prior to participation. The COnsolidated criteria for REporting Qualitative research (COREQ) checklist was used to guide reporting [18]. A completed COREQ checklist for this study is shown in Supplementary Material A.

### Participants and setting

Eligible participants were general practice staff with a potential role in the implementation of a text message-based support intervention for people with type 2 diabetes. This included healthcare professionals (e.g., GPs) and staff (e.g., receptionists) working in NHS general practices or affiliated with general practices (e.g., community pharmacists) in Manchester and Oxford. A previous study of a similar nature with 35 participants [19] was used for guidance on approximate sample size in advance of data collection. Recruitment of participants continued until the data collected was judged by the research team to be adequate in both amount and variety to answer the research question [20].

Potential practices for the recruitment of participants were identified through the UK Clinical Research Network in Manchester, and from lists of practices that had previously expressed interest in research at local diabetes-specific events in Oxford. Practices were sent a study invitation with details of the study and were asked to follow up by contacting the research team; either by phone, email or using a FREEPOST reply slip. Interested practices identified potential participants from within their staff who may have a role in the implementation of a text message-based support intervention. Potential participants were then sent the participant information sheet and consent forms by the research team.

### Procedure

Focus groups were carried out in Manchester by YKB, a female post-doctoral researcher with expertise in health psychology and qualitative methods, and observed by a male PhD student with an interest in intervention development and qualitative methods. Focus groups were conducted in Oxford by NN, a female digital health & wellbeing researcher with experience in qualitative methods, and VW, a female clinical academic from a nursing background with experience in qualitative methods and health services research. Focus groups were conducted in person in a general practice setting between November 2017 and February 2018. To check that no new significant insights would be identified from continued from data collection, additional individual interviews were carried out by LM, a female researcher with experience in public health, in July 2019.

Interviewers and focus group facilitators had no previous relationship with participants and began each session with a brief introduction to the research team and reasons for conducting the research. A summary of the proposed SuMMiT-D intervention was provided at the beginning of each session, and a topic guide (Supplementary Material B) of open-ended questions exploring how care for diabetes is currently delivered and how a text message-based system to support medication adherence for type 2 diabetes would work in practice was used to flexibly guide focus groups and interviews. This topic guide was developed with reference to Normalisation Process Theory [21, 22] to ensure breadth of coverage of relevant constructs including coherence (sense-making work around new working practices), cognitive participation (relational work to build and sustain a community of practice), collective action (operational work that people do to enact a set of practices) and reflexive monitoring (the appraisal work that people do to assess and understand the ways that a new set of practices affect them and others).

Only participants and interviewers were present during data collection, participants were encouraged to lead the conversation, and topics were followed up using non-directive general prompts. Conversations were audio-recorded, transcribed verbatim and checked for accuracy. Transcripts were not returned to participants for comment or correction. Providing participants with the opportunity to edit transcripts can lead to challenges in the context of focus groups; if some participants wish to change or add to the transcript, this may alter the context within which data were provided by other participants, leading to a less accurate representation of the focus group discussion. Transcripts were anonymised and imported into the software package NVivo version 12 to facilitate data organisation, management, and analysis.

### Data analysis

Data were analysed without trying to fit codes and themes into a pre-existing coding frame following an inductive reflexive thematic analysis approach [23]. Taking an open inductive approach, rather than using a pre-determined framework, allowed for flexibility in the analysis and allowed us to identify actions required for future implementation and any requirements for further development of the intervention to make it appealing to key general practice stakeholders. Analysis was conducted from a subtle realist perspective; subtle realism acknowledges the subjective nature of knowledge while maintaining a belief in the existence of an underlying reality that we attempt to represent through research [24]. Data analysis was conducted by KB, a Health Psychology MSc student, and JMS, a health psychologist and experienced qualitative researcher, and informed by discussion with the wider research team.

Analysis began with familiarisation, and KB read and re-read transcripts, whilst simultaneously logging relevant observations and potential code names. Once familiarisation was complete, KB proceeded to code the transcripts inductively in NVivo, coding each transcript line-by-line. Initial coding was reviewed by JMS and discussed. Following this discussion, KB collected existing codes into potential themes in NVivo using mind maps. KB used narrative summaries to explore different interpretations of the data and possible links between codes and themes. A draft was sent to JMS for review, and a second meeting was arranged to discuss theme development. KB continued by refining the themes and constructing a cohesive narrative. KB then identified and incorporated relevant quotes to support the analysis. Throughout this process, KB wrote reflexive logs to document the process of code and theme development [25]. Participants did not provide feedback on the findings.

## Results

Forty-one participants took part in seven focus groups. Participants included GPs (*n* = 16), pharmacists (*n* = 6), nurses (*n* = 5) and receptionists (*n* = 3) amongst others. For a full breakdown of participants per focus group see Supplementary Material C. Focus groups had a mean duration of 37 min (range 20–60 min). Five participants took part in interviews and included GPs (*n* = 2) and research nurses (*n* = 3) with a mean interview length of 16 min (range 13–21 min).

Five themes were developed. *The potential of technology as a patient ally* outlines a general need for diabetes support and conveys staff receptiveness to using technology to make this support more readily available. *Limited resources and assigning responsibility* and *Treating the patient: more than diabetes medication adherence* describe potential challenges to implementation, particularly as part of routine care. *Selling the intervention: what do general practice staff need to see?* and *Fitting the mould: complementing current service delivery* describe how implementation of the SuMMiT-D intervention can be supported and encouraged, and how challenges may be overcome to achieve better outcomes. Themes are described below and illustrated with participant quotations, each of which is accompanied by a focus group (FG)/interview number and details of the type of participant and participant number.

### Theme 1: the potential of technology as a patient ally

The need for support for people with type 2 diabetes was universally acknowledged. Although participants described how patients “almost want someone to hold their hand” (FG3, Clinical Pharmacist 2), resource constraints often hindered the ability of staff to meet these needs. This fuelled optimism about a text message intervention, with participants viewing technology as a means to bridge this gap. Participants felt additional support would be particularly valuable in disseminating information to people with type 2 diabetes as participants recognised that people with type 2 diabetes often become overwhelmed by the volume of information presented during consultations.

*“I was actually aware that I was giving him loads of information, and I didn't even get onto whether or not he needed medication [laughing].” (FG5, Practice Nurse1)*

In the absence of appropriate supports, participants were concerned about patient reliance on unreliable, external sources which perpetuate misconceptions surrounding medication use and side effects. As such, participants felt that poor medication adherence was rooted in a poor understanding of both diabetes and the medication used to treat it. A text message-based intervention to support medication use was perceived as being a reliable information source which could support people with type 2 diabetes by introducing and reinforcing information on a more gradual basis.

*“Part of my selling for it would be more along the lines of ‘there is an awful lot to take in, information…don’t panic because you’re going to get these little reminders and it will just – you’ll learn as you go along.’” (FG6, GP1)*

Participants also suggested that the potential to provide consistent prompts through text messages may empower people with type 2 diabetes to become more involved in their own treatment. It was hoped that by providing support in this way, people would become intrinsically motivated to take medication as prescribed.

*“I think it could help, because it's an extra thing to try and get the patients to engage, and look after their own health, and it might motivate them more.” (FG3, GP2)*

However, participants were also conscious that technology may not be an appropriate support for those who are not tech-literate or who have additional needs.

*“…realistically, there will be people for whom this isn’t suited. So, older, less technical savvy people, where, you know, language or even, and don’t forget, other impediments like vision, and hearing, as well, where it’s just not gonna be appropriate for them to use.” (FG3, GP1)*

### Theme 2: limited resources and assigning responsibility

Although participants recognised the potential of the SuMMiT-D intervention to address many of the gaps in current practice, they were apprehensive about implementation as part of routine care. Participants expressed the need for an external “support mechanism” (FG2, Pharmacist1) to provide training and additional supports and feared such resources may not be available outside of a research context. GPs were conflicted as they could not be as involved as they would like due to their excessive workloads. Whilst they felt it was important to monitor the texts being received by their patients, they also felt they didn’t have the resources available to make that level of commitment.

*“…we’re already overwhelmed with documentation and stuff, I think it would just swamp us…in terms of routinely showing that information, we’d have to say, no thanks” (FG3, GP1)*

Time constraints were a major issue discussed by GPs and nurses in relation to their involvement, with many worrying that introducing the intervention and recruiting people with type 2 diabetes would not be possible during time-limited consultations.

*“Oh crikey – you know – I’m already running late with this consultation, if you’re going to ask me to do something else that takes even another two minutes, that’s twenty percent of my consultation time.” (FG5, Practice Nurse2)*

GPs and nurses were not just concerned about their own workload, but also about that of their support staff. Participants felt that it would be unreasonable to expect receptionists to assist people with type 2 diabetes in opting-in to the intervention, since they are already overworked.

*“I’m really, really nervous about anything that takes away stuff from our staff.” (FG5, Practice Nurse1)*

As a result, some GPs advocated away from implementation of the SuMMiT-D intervention within general practice entirely, suggesting a greater role for pharmacists and that the role of the practice should be limited to signposting.

*“I mean, the question therefore belies all this as to whether primary care as it exists now is the right model to use this, kind of, technology or whether it is in fact pharmacies…” (FG1, GP1)*

Pharmacy staff also felt overworked and were similarly reluctant to take on additional obligation for implementing a new intervention.

*“Can I completely outright advocate away from pharmacists, because we do enough. [all laugh]” (FG2, Pharmacist1)*

However, pharmacists’ concerns were not solely rooted in workload. Pharmacy staff felt people with type 2 diabetes may be less receptive to the intervention if it were initiated in pharmacies, outlining that people can be sceptical of pharmacists’ motives, whereas GPs are viewed as being more objective. As a result, pharmacists felt their role should be limited to support and promotion, whilst GPs should be responsible for initiating the intervention.

*“Much better received from the GP. If we start…I’ve always found trying to start something from our end is much harder…I think it has to be from the top, from them and then down to us, and then we can still support it, promote it for those that might have been missed.” (FG2, Pharmacist1)*

Although there was lack of agreement about the extent of involvement of different healthcare professionals, all participants were receptive to supporting implementation, recognising that success of the intervention would require a collaborative approach.

*“I think whatever you do, you're going to need to like triangulate multiple times to try and get one person to actually do it… you know, sort of every single level of healthcare they go through, they get advised of this service.” (FG6, GP2)*

However, participants stressed that their role in the intervention needed to be as simple as possible, to reduce burden and allow them to balance other obligations.

*“Our role in it would have to be quite simple, in the way that we – Signposting. Or give them a leaflet or something.” (FG5, GP1)*

Participants also agreed that there was a need to provide a clear outline of the roles and responsibilities of various healthcare professionals at the outset, to allow all parties to make an informed decision about their involvement.

*“…if they don’t understand the sort of synopsis really of what’s required from the patient, or required from the practice, then they’re nervous about raising it with patients” (Interview 1, GP)*

### Theme 3: selling the intervention; what do general practice staff need to see?

Although there was reluctance amongst participants to assume responsibility for the SuMMiT-D intervention, several key features were identified which would make them more inclined to do so. Participants felt it was extremely important that the intervention attempt to address language and cultural barriers. Despite representing a large proportion of the type 2 diabetes population, participants expressed difficulties in supporting the South Asian community. Whilst part of this was attributed to language difficulties, participants suggested that communication issues are likely compounded by “a load of cultural stuff that [we] don't probably get” (FG5, Practice Nurse1). Participants described that to stand out as a valuable asset, the SuMMiT-D intervention must attempt to remedy these issues. They also stressed that the intervention should move beyond directly translating existing messages and address medication adherence from varied cultural perspectives.

*“…I think you would need to really carefully address the cultural issues, you shouldn't just translate from English. There needs to be a whole other thing about it.” (FG5, Practice Nurse2)*

Incentivisation was also raised as an important consideration. Participants articulated that some of their reservations surrounding the SuMMiT-D intervention related to the potential loss of incentives, as the intervention could be viewed as a competitor to their own local systems.

*“…there's a lot of pressure on us to have more of our patients using our online access…we'll be penalised if not many of our—not enough of our patients are doing that. So, we're going to be—and I imagine all GP practices are going to be quite nervous about signing up to anything that might dissuade our patients [laugh] from using our own thing. Because actually we need them to use our own thing.” (FG5, Practice Nurse2)*

Financial incentives were identified as a key motivator, with GPs expressing a tendency to prioritise innovations that are financially beneficial to the practice.

*“…you have to think in terms of how you're going to make money out of this; it's how GPs think.” (FG7, GP1)*

However, the focus was not solely on financial incentives; effectiveness and improved outcomes were also key motivators.

*“But the most important thing is it's improving outcomes from patients, isn't it? The quality of care that we're giving.” (FG7, Diabetes Nurse1)*

Participants described that for the intervention to be sustained, it must demonstrate measurable benefits, both in terms of feedback from people with type 2 diabetes and objective outcomes, which could be observed at a local level.

*“I think the only way you could do that is to do an audit now, look at compliance, and then after we’ve introduced this system [agreement in background] revisit the same patients…that’s the only scientific way to do it.” (FG4, GP2)*

### Theme 4: fitting the mould; complementing current service delivery

Participants described how the integration of the SuMMiT-D intervention into routine care would be largely dependent on the intervention’s ability to complement current service delivery. Participants felt that the intervention should fit seamlessly into their routine to minimise burden.

*“…it’s not an extra job, but just part of getting onto the next patient.” (FG7, GP1)*

Participants felt to facilitate implementation that the technology underpinning the intervention must be compatible with programmes already used within the practice.

*“Physically, setting it up, how is it gonna sit with our systems?” (FG3, GP3)*

GPs used the example of pop-ups, which use patients’ files to provide electronic prompts to practitioners during consultations, to demonstrate how integration may be achieved. Features such as pop-ups, which are already familiar due to their widespread use in other contexts, were described as a potential acceptable approach to integration. However, participants also stressed the importance of ease of use, emphasising that the intervention should require minimal interaction to prevent intrusion during consultations.

*“I would like to make sure that there are not too many clicks… because you’re doing other things and so you don’t want to be unnecessarily distracted from what you’re doing…” (Interview 2, GP)*

The importance of complementing existing practices was also raised in relation to message content. Participants were clear that the role of the intervention should be to supplement and enhance existing practices, and that it is “not a substitute to the care that [patients] already receive” (FG3, GP1). Consequently, healthcare providers felt that advice offered by text message should be consistent with that obtained from healthcare professionals. It was suggested that to ensure consistency, text messages sent as part of the SuMMiT-D intervention should be further developed with existing healthcare guidelines in mind.

*“As long as you’re using, like, Diabetes UK, and NICE guidelines, and things like that, that are recognised, then there shouldn’t be an issue.” (FG3, GP2)*

### Theme 5: treating and supporting the patient; more than diabetes medication adherence

The main aim of the SuMMiT-D intervention is to support medication adherence in type 2 diabetes; however, this narrow scope was a source of concern for some participants. General practice treats people holistically, rather than focusing just on individual conditions, and it was suggested that the SuMMiT-D intervention may be at odds with this philosophy.

*“…it would have to be useful to us, to be useful to our patients who we are trained not to, kind of, treat as a condition, but holistically. So, my diabetic patients are diabetic, but they have other things that, as we know, are wrong with them as well.” (FG1, GP1)*

Participants were uneasy about implementing an intervention focused solely on type 2 diabetes, when diabetes is almost always accompanied by other conditions.

*“Most type 2 diabetics don’t just have type 2 diabetes, they will always have something else with it.” (FG3, Practice Nurse1)*

Participants expressed reservations regarding the long-term sustainability of a single condition approach. Participants described the potential of mutually exclusive interventions being developed for all health conditions, highlighting that this would result in high confusion and low engagement, ultimately hindering service delivery.

*“…I just don’t want to also isolate the diabetics as technology can be useful for other long-term condition management. So, what would that…how would that affect my, our system as a whole? So, I’ve got this little, kind of, programme which just targets diabetics and this which targets my COPD patients and then I’ve got patients who are both diabetic and COPD …” (FG1, GP1)*

Participants also expressed concerned about text messages focusing solely on medication adherence and felt the scope ought to be expanded to include other aspects of self-management. Participants suggested the SuMMiT-D intervention should also include prompts addressing lifestyle factors such as diet and physical activity.

*“It’s not just medication prompting that they need, it’s the wider lifestyle issues that need addressing. So, it’s not enough to make sure you’re compliant with your medication. What’s the point if you’re eating chocolate biscuits every three hours, or whatever.” (FG3, GP1)*

Despite these reservations, a distinction was made between the intervention that would be used in a trial context and the system which would ultimately be adopted in general practice. Participants indicated that the intervention’s narrow focus may be acceptable in the interest of testing effectiveness in the short-term, so long as it is amenable to continued development over time.

*“I think it’s really important to limit the scope of the app to start with, you know…. I think as time goes on, you could add in the more diabetes lifestyle stuff…” (FG3, GP1)*

## Discussion

Five themes were developed based on general staff perceptions of the implementation of a text message intervention to support medication adherence in type 2 diabetes. The findings illustrate that whilst there is an appetite for additional support for people with type 2 diabetes (*Theme 1: the potential of technology as a patient ally*), potential implementation is impacted by a lack of both material and cognitive resources. Constraints such as time pressure, excessive workloads and case complexity mean general practice staff are reluctant to assume responsibility for implementation (*Theme 2: limited resources and assigning responsibility*). Despite these reservations, the findings suggest steps can be taken to increase the likelihood of practitioner involvement, such as situating new innovations within existing practices (*Theme 4: fitting the mould; complementing current service delivery*) and adding features which will appeal to healthcare staff (*Theme 3: selling the intervention: what do general practice staff need to see?*). Ultimately, participants felt that a text message-based intervention should be tailored to the needs of general practice patients and evolve to remain relevant (*Theme 5: treating patients; more than diabetes medication adherence*).

General practice staff clearly perceived a need to provide people with type 2 diabetes with more consistent medication adherence support. Health literacy was a prominent concern, with unreliable data sources and medication misconceptions seen to pose a considerable risk to medication adherence, consistent with previous literature [7]. Text message interventions were considered an acceptable means of addressing these concerns and boosting the motivation of people with type 2 diabetes. An automatic text message-based system is compatible with participants’ preference for interventions that require minimal input from healthcare staff and that can be continuously updated at low cost, based on evolving preferences and needs. However, participants expressed some concerns regarding accessibility, and emphasised that a text message intervention should supplement, rather than replace, existing care models to ensure all demographics continue to be catered for.

The findings highlighted a link between responsibility concerns and resource availability. Much like previous work in this field, researchers found staff were concerned about inadequate resources and organisational systems for implementation [26] and lacked clarity regarding the roles and responsibilities involved in implementing digital interventions [27]. These issues appear to be linked with resource scarceness exacerbating conflict regarding assigning responsibility. In the absence of clear policy and guidelines, some participants suggested assigning responsibility to other disciplines in an attempt to conserve their own resources. These conflicting agendas are a significant factor impeding collaboration across disciplines [27], resulting in a lack of shared commitments [28].

Participants stressed that to overcome these reservations, efforts need to be made to appeal to general practice staff both individually and collectively. Ease of use, incentivisation and the importance of complementing existing systems were identified as elements which would enhance workability and enable integration of the intervention into general practice. Interventions designed to target collective concerns, such as a holistic approach to care and catering for language and cultural diversity, may be beneficial in heightening shared commitment to implementation.

This study possesses several key strengths. One strength was the large sample size and inclusion of a range of general practice and affiliated pharmacy staff who could potentially become involved in implementation of the SuMMiT-D intervention in primary care. This allowed implementation to be considered in a variety of contexts, and for comparison across disciplines. The study also demonstrated the value of an implementation approach in the early stages of intervention development as the findings can be used to directly inform the further refinement of the SuMMiT-D intervention. For example, text messages supporting other self-management behaviours have now been developed for inclusion as part of the SuMMiT-D intervention, based on the preferences of healthcare staff and of people with type 2 diabetes [14] for diabetes management support beyond medication. The challenges to implementation identified will also be used to inform implementation strategies within the SuMMiT-D randomised trial evaluation, and as part of routine care if found to be effective.

A limitation of the study was the focus on anticipated implementation. Participants were provided with an overview of the SuMMiT-D intervention but did not have experience of interacting with the system, resulting in participants struggling at times to answer questions within the context of a hypothetical intervention. There may also be differences between how staff anticipate an intervention will be implemented, and how this would actually work in practice. The best approaches to considering implementation at various stages of development and evaluation of a new intervention and the benefits and challenges of qualitative research focused on anticipated versus actual implementation are worthy of further consideration.

A further limitation of the study is that the general practice staff who chose to take part may have had a particular interest in type 2 diabetes and in supporting medication adherence. Although recruitment of participants continued until the data collected was judged to be adequate to answer the research question with the available sample, additional insights may have been identified from a more diverse sample.

The study focused on implementation of the SuMMiT-D intervention at the individual practice level, and on general practice staff views, with parallel work focusing on the perspectives of people with type 2 diabetes [14, 16]. However, implementation at scale also requires buy-in from healthcare funders who make decisions on the integration of new approaches into routine care. Future studies should consider a whole systems approach and combine the perspectives of healthcare commissioners and policy makers with the views of people with type 2 diabetes and healthcare professionals to facilitate a deeper understanding of the context of intervention implementation.

## Conclusions

People with diabetes need additional support to self-manage their health, including taking medications as prescribed. Automated text message-based interventions, such as the SuMMiT-D intervention, have the potential to address this need and to meet requirements that interventions complement existing care and place minimal burden on staff time and resources. For routine implementation, interventions also need to address general practice priorities, such as taking a holistic approach to care and having multi-cultural reach and relevance. This study highlights the value of integrating an implementation focused approach in the early stages of the intervention development and evaluation process to inform both further intervention refinement and plans for implementation.

## Supplementary Information

**Additional file 1: Supplementary Material A.** COnsolidated criteria for REporting Qualitative research (COREQ) Checklist. **Supplementary Material B.** Topic Guide.** Supplementary Material C.** Characteristics of Focus Group Participants (*n* = 41).

## Data Availability

The datasets generated and analysed during the current study are not available as consent was not obtained from participants for transcripts to be made available.

## Abbreviations

COREQ: COnsolidated criteria for REporting Qualitative research
SuMMiT-D: The SUpport through Mobile Messaging and digital health Technology for Diabetes (SuMMiT-D)
GPs: General Practitioners
