# Supplementary material for: Implementing a text message-based intervention to support type 2 diabetes medication adherence in primary care: a qualitative study with general practice staff
Source: BMC Health Serv Res. 2023 Jun 10;23:614. doi: 10.1186/s12913-023-09571-9 (PMC10257158; doi:10.1186/s12913-023-09571-9)
Supplement: Supplementary file 1 — Additional file 1: Supplementary Material A. COnsolidated criteria for REporting Qualitative research (COREQ) Checklist. Supplementary Material B. Topic Guide. Supplementary Material C. Characteristics of Focus Group Participants (n = 41). [file 12913_2023_9571_MOESM1_ESM.docx]

# **Supplementary Material**

**Supplementary Material A: COnsolidated criteria for REporting Qualitative research (COREQ) Checklist**

| **No. Item** | **Guide questions/description** | **Reported on Page #** |
| --- | --- | --- |
| **Domain 1: Research team and reﬂexivity** | | |
| Personal Characteristics |  |  |
| 1. Interviewer/facilitator | Which author/s conducted the interview or focus group? | 4 |
| 2. Credentials | What were the researcher’s credentials? E.g., PhD, MD | 4 |
| 3. Occupation | What was their occupation at the time of the study? | 4 |
| 4. Gender | Was the researcher male or female? | 4 |
| 5. Experience and training | What experience or training did the researcher have? | 4 |
| Relationship with participants |  |  |
| 6. Relationship established | Was a relationship established prior to study commencement? | 4 |
| 7. Participant knowledge of the interviewer | What did the participants know about the researcher? e.g., personal goals, reasons for doing the research | 4 |
| 8. Interviewer characteristics | What characteristics were reported about the inter viewer/facilitator? e.g., Bias, assumptions, reasons and interests in the research topic | 4 |
| **Domain 2: study design** |  |  |
| Theoretical framework |  |  |
| 9. Methodological orientation and Theory | What methodological orientation was stated to underpin the study? e.g., grounded theory, discourse analysis, ethnography, phenomenology, content analysis | 5 |
| Participant selection |  |  |
| 10. Sampling | How were participants selected? e.g., purposive, convenience, consecutive, snowball | 3 |
| 11. Method of approach | How were participants approached? e.g., face-to-face, telephone, mail, email | 3 |
| 12. Sample size | How many participants were in the study? | 5-6 |
| 13. Non-participation | How many people refused to participate or dropped out? Reasons? | 5-6 |
| Setting |  |  |
| 14. Setting of data collection | Where was the data collected? e.g., home, clinic, workplace | 4 |
| 15. Presence of non-participants | Was anyone else present besides the participants and researchers? | 4 |
| 16. Description of sample | What are the important characteristics of the sample? e.g., demographic data, date | 5-6  Supplementary Material C |
| Data collection |  |  |
| 17. Interview guide | Were questions, prompts, guides provided by the authors? Was it pilot tested? | 4  Supplementary Material B |
| 18. Repeat interviews | Were repeat interviews carried out? If yes, how many? | N/A |
| 19. Audio/visual recording | Did the research use audio or visual recording to collect the data? | 4 |
| 20. Field notes | Were ﬁeld notes made during and/or after the interview or focus group? | 5 |
| 21. Duration | What was the duration of the interviews or focus group? | 5-6 |
| 22. Data saturation | Was data saturation discussed? | 3 |
| 23. Transcripts returned | Were transcripts returned to participants for comment and/or correction? | 4 |
| **Domain 3: analysis and ﬁndings** |  |  |
| Data analysis |  |  |
| 24. Number of data coders | How many data coders coded the data? | 5 |
| 25. Description of the coding tree | Did authors provide a description of the coding tree? | N/A |
| 26. Derivation of themes | Were themes identiﬁed in advance or derived from the data? | 5 |
| 27. Software | What software, if applicable, was used to manage the data? | 5 |
| 28. Participant checking | Did participants provide feedback on the ﬁndings? | 5 |
| Reporting |  |  |
| 29. Quotations presented | Were participant quotations presented to illustrate the themes/ﬁndings? Was each quotation identiﬁed? e.g., participant number | 6-14 |
| 30. Data and ﬁndings consistent | Was there consistency between the data presented and the ﬁndings? | 6-14 |
| 31. Clarity of major themes | Were major themes clearly presented in the ﬁndings? | 6-14 |
| 32. Clarity of minor themes | Is there a description of diverse cases or discussion of minor themes? | 6-14 |

**Supplementary Material B: Topic Guide**

**Support through Mobile Messaging and Digital Health Technologies for Diabetes (SuMMiT-D)**

**Understanding how the intervention will be implemented**

**Briefing:**

**1) Thank participant for agreeing to take part.**

**2) Introduce self.**

**3) As described in the Participant Information Sheet we are interested in** finding out how care for medicine use is currently delivered and how a technology system to support taking medications for type 2 diabetes medications would work in practice.

**4) If at any time during the interview you do not wish to answer a question that’s okay.**

**5) I would like to record our conversation. The recording will be typed out, but everything you say will be anonymous. Your name and any names or places you mention will be taken out, so that if someone read your interview/focus group they would not know who you are.**

**6) If, at any stage, you wish to stop the audio recorder, please let me know.**

**7) Do you have any questions?**

**Topics to be explored: Below is a list of questions to be discussed in this study. The work will remain flexible with respect to participants’ agendas but we will cover the main topics outlined below. It is common in semi-structured work to develop topics and questions as new ideas emerge from early data collection. Therefore, we may add new topics as the interviews progress and data collection continues. However, the key topic of exploring how care for medicine use is currently delivered and how a technology system to support taking medications for type 2 diabetes medications would work in practice will remain the focus of the interview/focus group.**

1. **Current delivery of care for medication use**
   - What is your experience of delivering care for medication use in type 2 diabetes?
   - How do you interact with other professionals and people with diabetes in the delivery of care?
2. **Overall impressions of, and barriers and facilitators to, the implementation of a technology system to support taking medications for type 2 diabetes medications**

- What is your overall impression of this type of system-would it be useful or a hindrance to your work?
- What are the challenges to the implementation of this type of system?
- What would make it easier for the system to be implemented?

1. **Coherence: How staff individually and collectively understand and make sense of the new system**
   - How would a technology system to support taking medications for type 2 diabetes medications work in your healthcare setting?
   - What would you need to do to implement this system?
   - How does this compare to the current delivery of care for medication use?
2. **Cognitive Participation: How staff build and sustain engagement with the new system**
   - What would be the first steps in setting up a new technology system?
   - What would encourage you to initially engage with a new technology system?
   - What would motivate you to continue?
   - How would the new system be sustained?
3. **Collective Action: How people enact the new system**
   - How would you manage a new system?
   - How would you need to interact with other healthcare professionals?
   - How would the new system be organised and structured?
4. **Reflexive Monitoring: How people formally and informally appraise and understand the effects of the new system**
   - How would you decide if the new system is successful?
   - At a practice level, how would it be decided if the new system was worthwhile?
   - Do you think this would change over time?
5. **Summary**

- Was there anything I left out?
- Anything else you would like to tell me?

**Supplementary Material C: Characteristics of Focus Group Participants (n = 41)**

| Focus Group Number (Location) | Occupation |
| --- | --- |
| 1 (Manchester)  n = 4 | GP  Healthcare Support Worker  Medical Student  Practice Nurse |
| 2 (Manchester)   n = 11 | Accuracy Checking Technician  7 Dispensary Assistants  3 Pharmacists |
| 3 (Manchester)   n = 8 | 4 GPs  2 Clinical Pharmacists  Trainee Pharmacist  Practice Nurse |
| 4 (Manchester)   n = 7 | 4 GPs  3 Receptionists |
| 5 (Oxford)   n = 3 | 2 Diabetes Nurses  GP |
| 6 (Oxford)   n = 5 | 5 GPs |
| 7 (Oxford)   n = 3 | Diabetes Nurse  GP  Health and Research Practitioner |
